# The Rat Mammary Gland as a Novel Site of Expression of Melanin-Concentrating Hormone Receptor 1 mRNA and Its Protein Immunoreactivity

**DOI:** 10.3389/fendo.2020.00463

**Published:** 2020-07-31

**Authors:** Daniella S. Battagello, Aline R. Lorenzon, Giovanne B. Diniz, Lívia C. Motta-Teixeira, Marianne O. Klein, Jozélia G. P. Ferreira, Carlos M. Arias, Antoine Adamantidis, Luciane V. Sita, José Cipolla-Neto, Estela M. A. F. Bevilacqua, Paul E. Sawchenko, Jackson C. Bittencourt

**Affiliations:** ^1^Instituto de Psicologia, Nucleo de Neurociencias e Comportamento, Universidade de São Paulo, São Paulo, Brazil; ^2^Instituto de Ciencias Biomedicas, Laboratorio de Neuroanatomia Quimica, Universidade de São Paulo, São Paulo, Brazil; ^3^Departmento de Biologia Celular e Do Desenvolvimento, Instituto de Ciencias Biomedicas, Universidade de São Paulo, São Paulo, Brazil; ^4^Departmento de Fisiologia e Biofisica, Instituto de Ciencias Biomedicas, Universidade de São Paulo, São Paulo, Brazil; ^5^Laboratory of Neuronal Structure and Function, The Salk Institute for Biological Studies, La Jolla, CA, United States; ^6^Department of Neurology, Inselspital, University of Bern, Bern, Switzerland

**Keywords:** lactation, MCHR1, GPCR, neuroendocrinology, peptides

## Abstract

Lactation is a complex physiological process, depending on orchestrated central and peripheral events, including substantial brain plasticity. Among these events is a novel expression of pro-melanin-concentrating hormone (*Pmch*) mRNA in the rodent hypothalamus, such as the ventral part of the medial preoptic area (vmMPOA). This expression reaches its highest levels around postpartum day 19 (PPD19), when dams transition from lactation to the weaning period. The appearance of this lactation-related *Pmch* expression occurs simultaneously with the presence of one of the *Pmch* products, melanin-concentrating hormone (MCH), in the serum. Given the relevance of the MPOA to maternal physiology and the contemporaneity between *Pmch* expression in this structure and the weaning period, we hypothesized that MCH has a role in the termination of lactation, acting as a mediator between central and peripheral changes. To test this, we investigated the presence of the MCH receptor 1 (MCHR1) and its gene expression in the mammary gland of female rats in different stages of the reproductive cycle. To that end, *in situ* hybridization, RT-PCR, RT-qPCR, nucleotide sequencing, immunohistochemistry, and Western blotting were employed. Although *Mchr1* expression was detected in the epidermis and dermis of both diestrus and lactating rats, parenchymal expression was exclusively found in the functional mammary gland of lactating rats. The expression of *Mchr1* mRNA oscillated through the lactation period and reached its maximum in PPD19 dams. Presence of MCHR1 was confirmed with immunohistochemistry with preferential location of MCHR1 immunoreactive cells in the alveolar secretory cells. As was the case for gene expression, the MCHR1 protein levels were significantly higher in PPD19 than in other groups. Our data demonstrate the presence of an anatomical basis for the participation of MCH peptidergic system on the control of lactation through the mammary gland, suggesting that MCH could modulate a prolactation action in early postpartum days and the opposite role at the end of the lactation.

## Introduction

The melanin-concentrating hormone (MCH) peptidergic system was first characterized in mammalian species in 1989 (1, 2) and mapped in the rat central nervous system (CNS) for the first time in 1992 (3). Although the tuberal hypothalamus harbors the largest expression of the pro-melanin-concentrating hormone (*Pmch*) mRNA and peptide in both sexes (3, 4), transient expression of *Pmch* mRNA is observed in the preoptic hypothalamus of lactating females (5). This novel expression (and peptide synthesis) encompasses the ventromedial aspect of the medial preoptic nucleus (vmMPOA), the periventricular preoptic nucleus, and the most rostral aspects of the paraventricular nucleus of the hypothalamus with peak expression occurring on postpartum day 19 (PPD19) (5–10).

Although no colocalization between MCH and oxytocin (OT) is found in the paraventricular nucleus, MCH-immunoreactive (MCH-ir), and OT-ir fibers travel through the internal lamina of the median eminence in close apposition (9). These MCH-ir fibers then reach the neurohypophysis, where MCH is presumptively released into the bloodstream (9). Despite overwhelming evidence of hormonal release of MCH in mammals (11–16), it is unclear what physiological role does it play or what is its relevance for lactation and weaning. One possible strategy to elucidate these questions is to identify targets of MCH action in the periphery that are contextually related to the lactation period.

Melanin-concentrating hormone acts through two different G protein-coupled receptors (GPCRs), MCHR1 (17–21) and MCHR2 (22–27). Although MCHR1 is ubiquitously expressed in vertebrates, MCHR2 has been lost in the *Glires* clade, including rabbits and rodents (28, 29). In addition to the CNS (10, 30), MCHR1 has been reported in several peripheral tissues, including eye, skeletal muscle, intestine, adipose tissue, placenta, and bone (20, 23). However, to the best of our knowledge, the mammary gland has not been examined for the presence of MCHR1.

The mammary gland is a complex secretory structure, exclusively found in the class Mammalia, which consists of a parenchyma and adipose stroma, respectively derived from ectoderm and mesoderm (31–34). Male and female rat mammary glands present six pairs of glands along the milk line and sexual dimorphism with specific differences in their morphologies [for review, see (33)]. Under specific hormonal influence, the female rat mammary gland develops the secretory epithelium (32) during pregnancy, which is responsible for milk production. After weaning, there is a mammary gland involution followed by alterations that decrease the expression of milk proteins and promote the return of the glandular tissue to its prepregnancy state (34).

The peak synthesis of MCH in the hypothalamus and of MCH-ir fibers in the ME near the weaning period, which accompanies the cessation of lactation and involution of mammary glands, puts MCH neurons in a unique position to bridge the central control of energy expenditure (35–37) and the periphery, fitting well our proposition that MCH's prime function is as a maintainer of the homeostatic baseline (38). Therefore, we hypothesized that MCHR1 can be found in the mammary gland, and its presence is dynamically modulated by the lactation stage of the dam.

## Materials and Methods

### Animals and Experimental Groups

Adult female and male Long-Evans rats (*n* = 76) were bred and raised in the animal facility of the department of anatomy (Universidade de São Paulo, Instituto de Ciencias Biomedicas) in a light- and temperature-controlled environment (12-h light–dark cycle, 22 ± 2°C) with standard food chow and tap water *ad libitum*. All procedures were performed in accordance with local regulations (39). The studies involving animal subjects were reviewed and approved by the Committee on Ethical Research on Animals of the Institute of Psychology (Protocol CEPA #003/2012) and by the Ethics Committee on Animal Use of the Institute of Biomedical Sciences (Protocol CEUA #035/2012).

When females reached 3 months of age (250–270 g), they were group housed in propylene cages, and the estrous cycle was verified daily (between 9 and 10 am) by vaginal cytology (40). After the confirmation of two regular estrous cycles, female virgins were randomly assigned to the diestrus group (*n* = 19), constituting the control group of non-lactating rats (diestrus). To form the lactating groups, randomly assigned female rats with two regular estrous cycles were mated with experienced male rats (*n* = 10) in the afternoon of proestrus. Pregnancy was confirmed by the presence of sperm in the vaginal cytology analysis the day after mating. Pregnant females were individually housed in propylene cages until the end of the experiments. The day of parturition was designated as PPD0, and the litters were culled to eight pups (four females and four males) at PPD2. The lactating groups (*n* = 47) were comprised of randomly assigned lactating dams at 5 (PPD5), 12 (PPD12), and 19 (PPD19) days after term. Within each group, subjects were randomly assigned to transcardiac perfusion or decapitation methods and euthanized at the appropriate day of lactation.

To ensure the antibody specificity, we used mammary gland tissue from *Mchr1* knockout mice (*Mchr1*^−/−^ mice) (41) at PPD19. These animals (*n* = 3) were bred and raised in the animal facility of the Inselspital University Hospital (Bern, Switzerland) in similar conditions as described above.

### Tissue Processing

#### Animal Perfusion and Tissue Preparation

Animals assigned to *in situ* hybridization protocol (ISH) received an excess of 35% chloral hydrate (1 mL i.p.) and were perfused transcardiacally via the ascending aorta with ~100 mL of cold 0.9% saline. This step was followed by 750 mL of cold 4% formaldehyde in borate buffer (0.1 M, pH 9.5) for 25 min.

Mammary gland pairs from the abdominal sector were carefully dissected, embedded in Tissue Tek® (Sakura Finitek, USA), and kept at −80°C. Samples were then cut in a cryostat (CM1850; Leica, Germany) in six series of sagittal sections (15-μm-thick) collected onto adhesion glass slides (Fisher Scientific, USA) and stored at −30°C until further processing (see below). For reference purposes, series of slices underwent hematoxylin-eosin staining.

#### Decapitation and Tissue Preparation

Animals assigned to PCR, nucleotide sequencing, Western blotting, and immunohistochemistry were anesthetized with an excess of 35% chloral hydrate (1 mL, i.p.) and euthanized by decapitation. For RNA extraction, the skin was carefully removed from the rest of the mammary gland in all four sectors (cervical, thoracic, abdominal, and inguinal). Each gland was then quickly dissected and cut into pieces ~5-mm-thick, washed in 0.2 M phosphate saline buffer (PBS, pH 7.4), carefully collected in tubes containing TRIzol® (Life Technologies Inc., USA), and frozen in dry ice. For protein extraction, pairs of mammary glands (abdominal sector) were quickly dissected, washed in PBS (0.2 M, pH 7.4), and collected in tubes followed by freezing the tissue in dry ice. All samples were kept at −80°C.

For immunohistochemistry, pairs of mammary glands (abdominal sector) were quickly dissected, washed in PBS, and carefully submerged in methacarn fixative solution (60% methanol, 30% chloroform, and 10% acetic acid) for 3 h at 4°C, which protocol was adapted from a previous study (42). Samples were washed in 100% alcohol and processed for paraffin embedding. Tissue sectioning was performed in a rotary microtome (American Optical, USA) to obtain sagittal 5-μm-thick slices. Slices were collected on positively charged glass slides (Knittel, Germany) and stored at room temperature (RT) until further processing.

### *In situ* Hybridization

The ISH procedure with ^35^S-labeled antisense cRNA probes was used to investigate and localize *Mchr1* mRNA*-*expressing cells in the mammary gland tissue (abdominal sector) of diestrus and lactating rats. To synthesize the *hMchr1* probes, a plasmid was designed by subcloning a 600-bp fragment comprising the coding region of h*Mchr1* into the *Bam*HI-X*ba*I sites of a pBluescript II SK(+) vector (Stratagene, USA), following protocol previously established (30). The RNA polymerases were applied to generate sense (T7) and antisense (T3) h*Mchr1* riboprobes. The protocol employed for ISH was adapted from previous work (43) while taking into consideration specific characteristics of the mammary tissue. Briefly, sections were fixed (4% formaldehyde in 0.1 M PBS) for 5 min, followed by protein digestion (0.05 M EDTA, 0.1 M Tris pH 8, 0.001% proteinase K) for 5 min at 37°C. Next, the samples were submitted to 0.25% acetylation for 10 min and washed in 2x saline sodium citrate (SSC). The samples were dehydrated, air dried, and then each slide received 100 μL of hybridization solution (50% formamide, 0.3 M NaCl, 0.01% SDS, 10 mM Tris (pH 8.0), 0.01% tRNA, 0.2% 5 M dithiothreitol, 1 mM EDTA pH 8.0, 1x Denhardt's solution, 10% dextran sulfate) containing the cRNA probes for *Mchr1* at 56°C overnight (≅18 h). Next, slices were submitted to 0.002% RNAse A treatment for 30 min in a 37°C water bath, followed by stringency washes. The tissue was dehydrated and air dried at RT. The slides were dipped in Kodak NTB-2, air-dried, and exposed at 4°C for 1 week. The slides were developed with Kodak D-19, counterstained with hematoxylin-eosin staining, dehydrated, cleared in xylene, and coverslipped with DPX (Sigma-Aldrich, USA).

### RNA Extraction, cDNA Synthesis, RT-PCR, and RT-qPCR

Mammary glands and hippocampus (used as a central nervous system positive control) were homogenized using TRIzol® in Precellys® 24 (BertinTechnologies, France). The samples' total mRNA was extracted using the Direct-zol™ Kit (Zymo Research Corp., USA). Quality and quantification of mRNA samples were determined by NanoDrop® (Fisher Scientific). The generation of cDNA occurred after treatment with DNase I (Life Technologies) through the use of a high-capacity cDNA kit (Life Technologies). Briefly, 1 μg of total RNA was treated with DNase I and incubated with kit buffers (10 min at 25°C, 120 min at 37°C and, 5 min at 85°C) in a thermocycler (Eppendorf, USA). The resulting cDNAs were used both for RT-PCR/RT-qPCR purposes.

Reverse transcriptase PCR was performed with Platinum® PCR SuperMix High Fidelity (Life Technologies) following these steps: 10 min at 95°C, 40 PCR cycles (1 min at 94°C, 90 s at 55°C, 90 s at 68°C) and 10 min at 72°C. The *Mchr1* and *Gapdh* products were visualized on a 1.5% agarose gel electrophoresis stained with ethidium bromide (Sigma-Aldrich).

Real-time qPCR was performed with SYBR® Green PCR Master Mix (Applied Biosystems, USA) with StepOnePlus (Applied Biosystems) under standard conditions: 10 min at 95°C, 40 qPCR cycles (15 s at 95°C, 1 min at 55°C), 15 s at 95°C, 1 min at 60°C, and 15 s at 95°C. The Ct values (triplicates) were analyzed using StepOnePlus Software 2.0 (Applied Biosystems). The amplification specificity was analyzed by the melting curve of the samples, and the 2^−ΔΔ*Ct*^ method (44) was used to quantify mRNA relative to the housekeeping endogenous control gene (*Gapdh*). Primers for *Mchr1* and *Gapdh* and amplification conditions for RT-PCR/RT-qPCR are listed in Table 1.

**Table 1:** Primers used in RT-PCR and RT-qPCR.

**Gene**	**Primers**	**Bases**	**Annealing**	**Cycles**	**PCR product**
***Mchr1***	**AS**: 5′- CAGGGTAGCCCTGGGTTTAAT-3′	21	55°C	40	639 pb
(RT-PCR)	**S**: 5′-GCGCTCTCCTTCATCAGTATC-3′	21			
***Mchr1***	**AS**: 5′- CTGACCTCTACTGGTTCACTCT−3′	22	55°C	40	102 pb
(RT-qPCR)	**S**: 5′-ACGTCATGCGCTGTAGTATTT−3′	21			
***Gapdh***	**AS**: 5′- TGGAAGATGGTGATGGGTTTC-3′	21	55°C	40	219 pb
(RT-PCR and RT-qPCR)	**S**: 5′-GGTCGGTGTGAACGGATTT−3′	19			

*AS, antisense; S, sense; bp, base pairs*.

### Nucleotide Sequencing

Amplified cDNA RT-PCR products (20 ng/μl) of *Mchr1* in the mammary gland (skin and non-skin) and hippocampus (PPD19 dam) were purified and concentrated using the illustra GFX PCR DNA, and Gel Band Purification Kit (GE Healthcare, UK) following the manufacturer's instructions. Samples were sequenced by automatic DNA sequencing from PCR products using the ABI 3730 DNA Analyzer (Applied Biosystems). Sequencing reactions were performed using the BigDye® Terminator v3.1 Cycle Sequencing Kit (Applied Biosystems). Quality control of reactions was performed using pGEM 3Zf (+) and primer M13 (-21). Sequences were analyzed by Sequencing Analysis 5.3.1 software using Base Caller KB, and the output files were analyzed and aligned with Chromas 2.4.4 (Technelysium, Australia) and MultAlin (45).

### Western Blotting

Mammary glands were homogenized using RIPA Lysis Buffer (50 mM Tris-HCl, 150 mM NaCl, 1% NP-40, 0.25% Na-deoxycholate, 0.1% SDS, 1 mM Na3VO4, 1 mM NaF, 1x Protease mix; Sigma Aldrich, Germany) in Precellys® 24 equipment (BertinTechnologies, France). The quantification of protein concentration was determined by the Bradford method following the manufacturer protocol (Sigma Aldrich). Briefly, samples (50 μg) were resolved by SDS-Page in 15% acrylamide gels (Bio-Rad, USA) and transferred to nitrocellulose membranes (0.45 μm, Bio-Rad) by using a trans-blot turbo transfer system (Bio-Rad, USA). Next, membranes were blocked in TBS-T-non-fat milk 5% (140 mM NaCl, 20 mM Tris-HCl pH 7.4, 0.1% Tween-20) for 6 h and incubated at 4°C with anti-MCHR1 polyclonal antibody (1:100, sc-5534) in TBS-T-nonfat milk 5% overnight. Then, membranes were washed three times with TBS-T buffer and incubated with secondary antibody, donkey anti-goat HRP (1:1000, Jackson ImmunoResearch, USA) in TBST-nonfat milk 2.5% for 1 h at RT and washed three times with TBS-T buffer.

After stripping, the same membranes were incubated with anti-β-Actin monoclonal antibody (1:1000, clone AC-15, Sigma) in TBS-T-nonfat milk 5% for 1 h at RT and then washed three times with TBS-T buffer. Finally, the membranes were incubated with secondary antibody, donkey anti-mouse HRP (1:1000, Jackson ImmunoResearch, USA) in TBS-T-nonfat milk 2.5% for 1 h at RT and washed three times with TBS-T buffer. Immunoreactive protein bands were revealed with Clarity™ Western ECL Substrate (Bio-Rad, USA), and images were acquired with an Amersham Imager 600 (GE Healthcare). Primary antibodies for MCHR1 and β-actin are listed in Table 2.

**Table 2:** Primary antibodies used in the experiments.

**Antibody**	**Manufacturer**	**Man. Code**	**Reference (First author, volume: pages, year and journal)**	**PubMed ID**	**RRID**	**Antigen sequence**	**Application/concentration**
Goat anti(human) melanin-concentrating hormone receptor 1, C-17 (anti-MCHR1)	Santa Cruz	sc-5534	Berbari NF, 105(11):4242–6, 2008—Proceedings of the National Academy of Sciences of the United States of America	18334641	AB_2143957	C-terminus of MCH-1R of human origin	1:100 [IF/WB]
Mouse anti(human) β-Actin (anti β-Actin)	Sigma	A5441	Rostoker R, 154(5):1701–10, 2018—Endocrinology	23515289	AB_476744	Slightly modified β-cytoplasmic actin N-terminal peptide, Ac-Asp-Asp-Asp-Ile-Ala-Ala-Leu-Val-Ile-Asp-Asn-Gly-Ser-Gly-Lys, conjugated to KLH	1:1,000 [WB]
Rabbit anti(human) melanin-concentrating hormone receptor 1 (anti-MCHR1)	Abcam	Ab97509	-	-	AB_10680290	Synthetic peptide corresponding to Human MCHR-1 aa 358–422.	1:100 [IF/WB]

*IF, immunofluorescence; WB, western blotting*.

### Immunohistochemistry

#### Antisera Characterization

In this work, two primary antibodies for MCHR1 (sc-5534, Santa Cruz Biotechnology, USA, and ab97059, Abcam, USA) were tested for the indirect immunofluorescence method; however, only sc-5534 showed consistent labeling specificity for MCHR1 cells in the mammary gland tissue. To determine the optimal labeling, we titrated the antibodies following a previously established protocol (46), and the omission of the primary and secondary antibodies were carried out. Moreover, to confirm the specificity of the antibodies, we also performed the same immunofluorescence method in the negative control tissue, the mammary gland tissue of *Mchr1*^−/−^ mice at PPD19. Primary antibodies for MCHR1 are listed in Table 2.

#### Immunofluorescence

Briefly, methacarn-fixed paraffin-embedded sections were submitted to antigen retrieval method in sodium citrate pH 6 (15 min in a 95°C water bath). Sections were rinsed in 0.02 M potassium phosphate buffer (KPBS, pH 7.4) and pretreated with a solution of 3% hydrogen peroxide diluted in KPBS for 5 min. Next, the sections were rinsed in KPBS and then incubated in KPBS solution containing 0.03% Triton X-100, 3% bovine serum albumin (blocking solution), and the polyclonal anti-MCHR1 antibody at a dilution of 1:100 (sc-5534) overnight at 4°C. Sections were washed in KPBS and incubated in KPBS solution containing 0.03% Triton X-100, and fluorophore-conjugated anti-IgG antibody at 1:200 (AlexaFluor 594, Molecular Probes, USA) for 2 h at RT. Next, sections were rinsed in KPBS, counterstained with 1:10,000 DAPI nuclear stain (Life Technologies) in KPBS for 15 min at RT, washed, air-dried, and coverslipped with Prolong Diamond Antifade Mountant (Life Technologies).

### Imaging and Data Analysis

Bright field and dark field photomicrographs were acquired with a DS-Ri1 digital camera (Nikon Corporation, Japan) coupled to an upright microscope (Leica) using the image capture software NIS-Elements BR 3.22 (Nikon Corporation). Wide field fluorescence photomicrographs were obtained with an AxioCam 506 (Carl Zeiss, Germany) coupled to an AxioImager Z2 motorized upright microscope with an HXP 120V illuminator (Carl Zeiss) and processed using the ZEN Blue Edition software (Carl Zeiss). All images were adjusted for brightness and contrast using Adobe Photoshop CS5.1 (Adobe Systems Inc., USA). The images of the bands of each gel obtained by the Western blotting were subjected to analysis by relative densitometry band quantification using ImageJ 1.52v software (47) and compared with the values obtained from β-actin (housekeeping protein).

Data were assessed for normality and homogeneity of variance to determine whether to use parametric or non-parametric statistical tests. All statistical analyses were performed using GraphPad Prism 8 software (GraphPad Software, USA). Outliers were identified and removed from the analysis through the Grubbs test. *Gapdh* and β-actin were used as normalizers for RT-qPCR and Western blotting, respectively. The results are presented as means ± S.E.M. Data from RT-qPCR and Western blotting experiments were evaluated through Mann–Whitney or Kruskal–Wallis non-parametric with multiple comparisons tests or one-way ANOVA followed by Tukey or Dunn's *post-hoc* when *p*-values reached significance. Statistical significance was set at *p* < 0.05.

## Results

### Cellular Localization of *Mchr1* mRNA in the Mammary Gland

**Figure 1:**
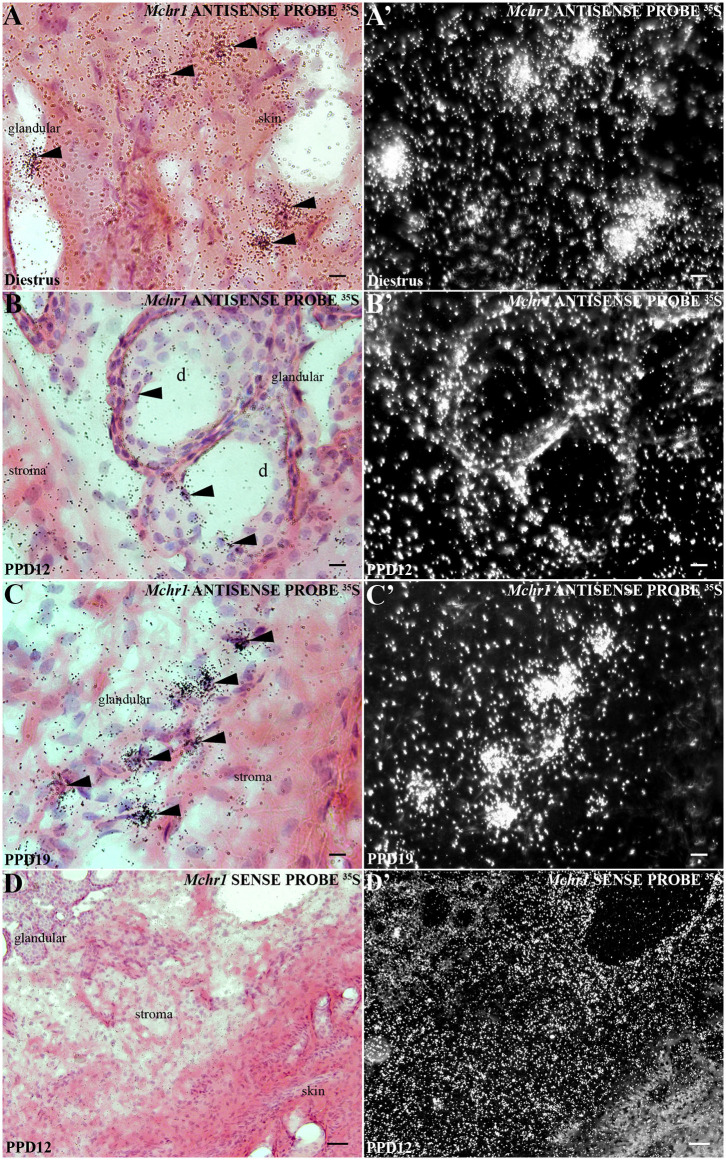
Distribution of *Mchr1* mRNA in the rat mammary gland tissue. A series of bright field photomicrographs showing *Mchr1* mRNA (silver grains) over hematoxylin-eosin-counterstained cells in the mammary gland tissue (skin and undifferentiated parenchyma) of **(A)** diestrus female rat, **(B)** PPD12, and **(C)** PPD19 dams, (*n* = 3/group). Dark field photomicrographs of **(A'- C')** display that the mammary gland tissue of diestrus female rat **(A')** presents a restricted *Mchr1* mRNA expression in skin and, the lactating dams in PPD12 **(B')** and PPD19 **(C')** present a parenchyma expression: *acini* and stroma. A series of bright field **(D)** and dark field **(D')** photomicrographs showing no labeled cells hybridized in the mammary gland tissue of lactating dam in PPD12 when cRNA *Mchr1* sense probe was applied. The *Mchr1* mRNA cells are indicated by black arrowheads. d, duct. Scale bars: 10 μm **(A–C)**, 10 μm **(A'–C')**, and 50 μm **(D, D')**.

*In situ* hybridization revealed an abundant presence of *Mchr1* mRNA in the mammary gland of female rats (Figure 1) with distinct patterns for non-lactating and lactating subjects. In diestrus female rats, silver grain deposits were exclusively found in the skin: epidermis and dermis (Figures 1A,A'). In the epidermis, silver grains preferentially deposited in the basal layer and in structures that invaginate into the dermis, such as hair follicles (not shown). In the dermis, *Mchr1*-expressing cells were identified on stromal cells juxtaposed to the basal layer of the epidermis, close to the skin attachments and in the connective and muscular tissue region (Figures 1A,A'). Although the morphology supports a fibroblast-like character for some of these labeled cells, other stromal cell types could not be identified. No evidence of *Mchr1* mRNA was found in the undeveloped glandular tissue.

In lactating animals, the epidermis and dermis were similarly labeled (Figures 1B,C). An additional site of silver grain deposition was detected in lactating animals: the glandular parenchyma. Cells labeled for *Mchr1* mRNA were found bordering ducts and *acini (alveoli*) (Figures 1B,B'). The hybridization signal for *Mchr1* was also found in the connective tissue that surrounds the mammary *alveoli* as well as in the connective tissue near the skin attachments and muscle tissue that surrounds the glandular part in contrast to the animals in diestrus phase (Figures 1C,C'). This *Mchr1* expression pattern was more evident in dams in PPD19 than PPD12. Control of the *in situ* hybridization technique was performed in adjacent section series. There was no *Mchr1* mRNA expression using *Mchr1* cRNA sense probe (Figures 1D,D').

### Differential mRNA Expression, Protein Variation, and Nucleotide Sequencing of *Mchr1* in the Rat Mammary Gland

**Figure 2:**
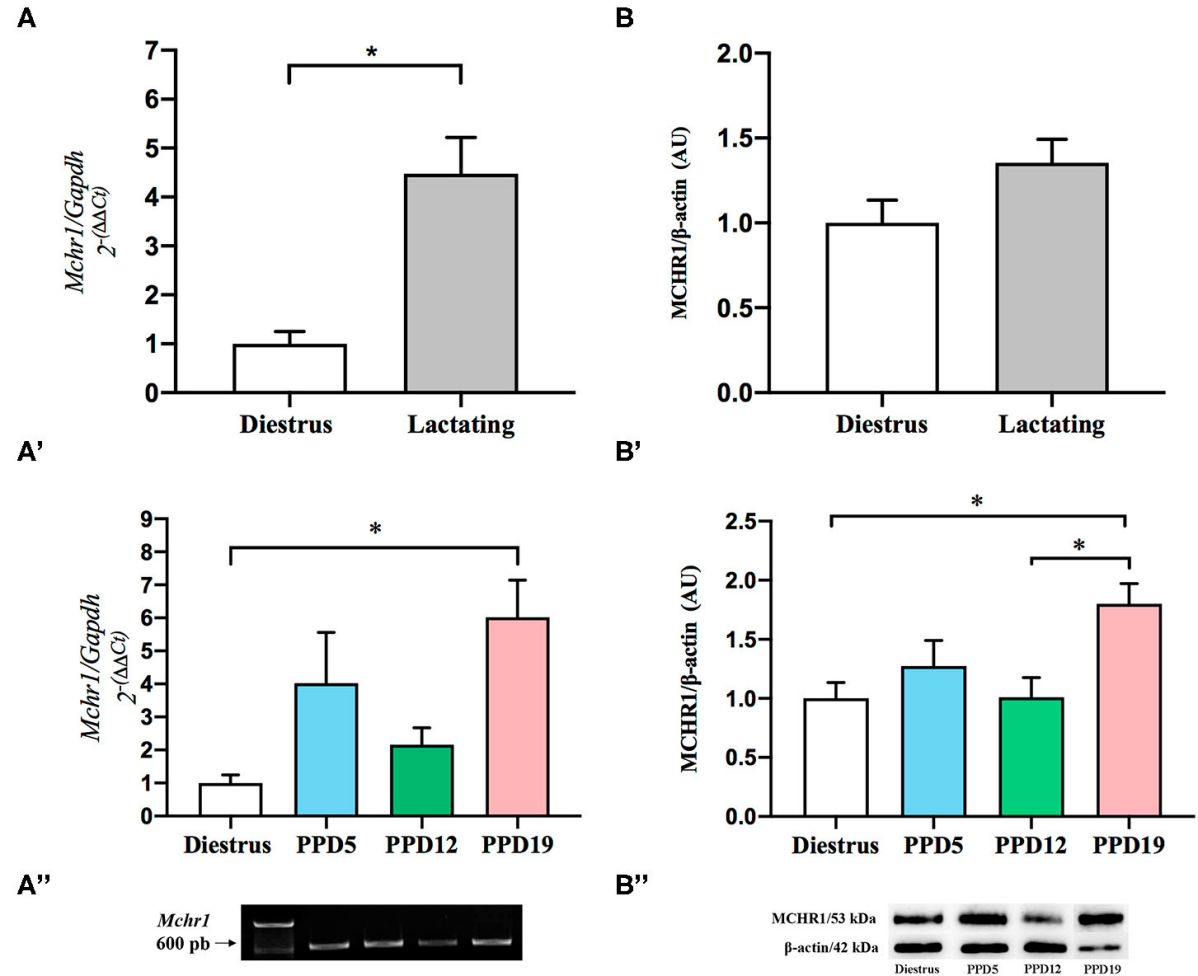
Effect of the lactation in the expression of mRNA *Mchr1* and MCHR1 protein content in the rat mammary gland. **(A)** Significant effect of lactation in the *Mchr1* expression in mammary gland of the lactating group when compared to the diestrus, *U* = 423, *p = 0.0002, Mann–Whitney. **(A')** Dunn's *post-hoc* analysis indicates differences between diestrus and PPD19, *K* = 19.37, *p = 0.0002, Kruskal–Wallis. **(A”)** Analysis of PCR products by agarose gel electrophoresis revealed the presence of *Mchr1* (639 bp) for mammary gland for all samples analyzed. Data are expressed as mean ± S.E.M. Diestrus (*n* = 8), PPD5 (*n* = 6), PPD12 (*n* = 5), PPD19 (*n* = 8). Relative *Mchr1* expression data were normalized using *Gapdh* as housekeeping gene. The final data presented in the graphics were normalized according to the diestrus value. **(B)** There was no significant difference in the MCHR1 protein level in the mammary gland of diestrus and lactating rats (*p* = 0.157), Student's *t*-test. **(B')** ANOVA revealed main group effect (*p* = 0.027) for different phases of the lactation period, with MCHR1 protein level increases with the progression of lactation; *p = 0.027. Tukey *post-hoc* analysis indicates a significant increase in the MCHR1 protein in PPD19 as compared to diestrus or PPD12 (*p = 0.027). **(B”)** Analysis of bands by SDS-Page in 15% acrylamide revealed the presence of MCHR1 (53 kDa) for mammary gland and the integrity with the presence of β-actin (42 kDa). Data are expressed as mean ± S.E.M. Diestrus (*n* = 5), PPD5 (*n* = 5), PPD12 (*n* = 4), PPD19 (*n* = 4). Relative MCHR1 protein levels were normalized using β-actin.

To quantify the apparent differences in the expression of *Mchr1* mRNA observed with ISH, we employed RT-qPCR. Grouped analysis of relative expression of *Mchr1* mRNA revealed that expression is significantly higher in the mammary gland of lactating dams (6.63 ± 1.09) when compared to diestrus (1.48 ± 0.36) *U* = 423, ^*^p = 0.0002, Mann–Whitney; Figure 2A). When divided into lactation subgroups, there is a significant main group effect, *K* = 19.37, ^*^p = 0.0002, Kruskal*-*Wallis), with Dunn's *post-hoc* analyses indicating a difference between diestrus and PPD19 (^*^p = 0.0002) (Figure 2A'). Analysis of PCR products of the representative groups by agarose gel electrophoresis revealed the presence of similar-sized *Mchr1* (639 bp) products in the mammary gland (Figure 2A”).

The Western blotting method was performed to provide more information about the amount of MCHR1 protein, considering the different stages of the reproductive cycle. There was no significant difference in the grouped analysis of MCHR1 protein level in the mammary gland of diestrus (0.91 ± 0.12) and lactating dams (1.23 ± 0.12); *t* = 1.485, *p* = 0.157, Student's *t*-test (Figure 2B). ANOVA revealed main group effect [*F*_(3, 14)_ = 4.135, *p* = 0.027] for different phases of the lactation period, MCHR1 protein level increases with the progression of lactation (Figure 2B'); Tukey *post-hoc* analysis indicates a significant increase in the MCHR1 protein in PPD19 as compared to female rats in diestrus or PPD12 (^*^p = 0.027) (Figure 2B'). Analysis of bands of the representative groups by SDS-Page in 15% acrylamide revealed the presence of MCHR1 (53 kDa) and β-Actin (42 kDa) in the mammary gland (Figure 2B”). Please see Supplementary Figure 1 for MCHR1 detection details by the Western blotting method.

Moreover, to investigate if there are nucleotide sequence alterations in the mammary gland *Mchr1* mRNA during lactation, we employed DNA sequencing method using cDNA PCR products of mammary gland and hippocampus (PPD19 dam). Analysis of aligned sequences demonstrated the existence of 100% sequence identity between mammary gland and its hippocampus *Mchr1* mRNA (Supplementary Figure 2).

### Cellular Localization of MCHR1 Immunoreactivity in the Mammary Gland

Immunohistochemistry was performed to confirm MCHR1 synthesis by cells in the mammary gland. However, to determine the specificity of the MCHR1 antibody used, we performed two sets of controls in both MCHR1 primary antibodies (sc5534 and ab97059): the use of *Mchr1*^−/−^ mice mammary gland tissue and omission of the primary antibodies. Reactions with ab97059 in knockout *Mchr1* tissue showed staining, demonstrating lack of specificity (Supplementary Figure 3A). However, reactions with sc5534 in knockout *Mchr1* tissue showed no staining (Supplementary Figure 3B). The suppression of the primary antibody sc5534 resulted in the absence of immunostaining for MCHR1 (Supplementary Figure 3C). These standard negative controls for antibody validation as recommended by previous study (48) showed that sc5534 primary antibody labels MCHR1 with high specificity.

**Figure 3:**
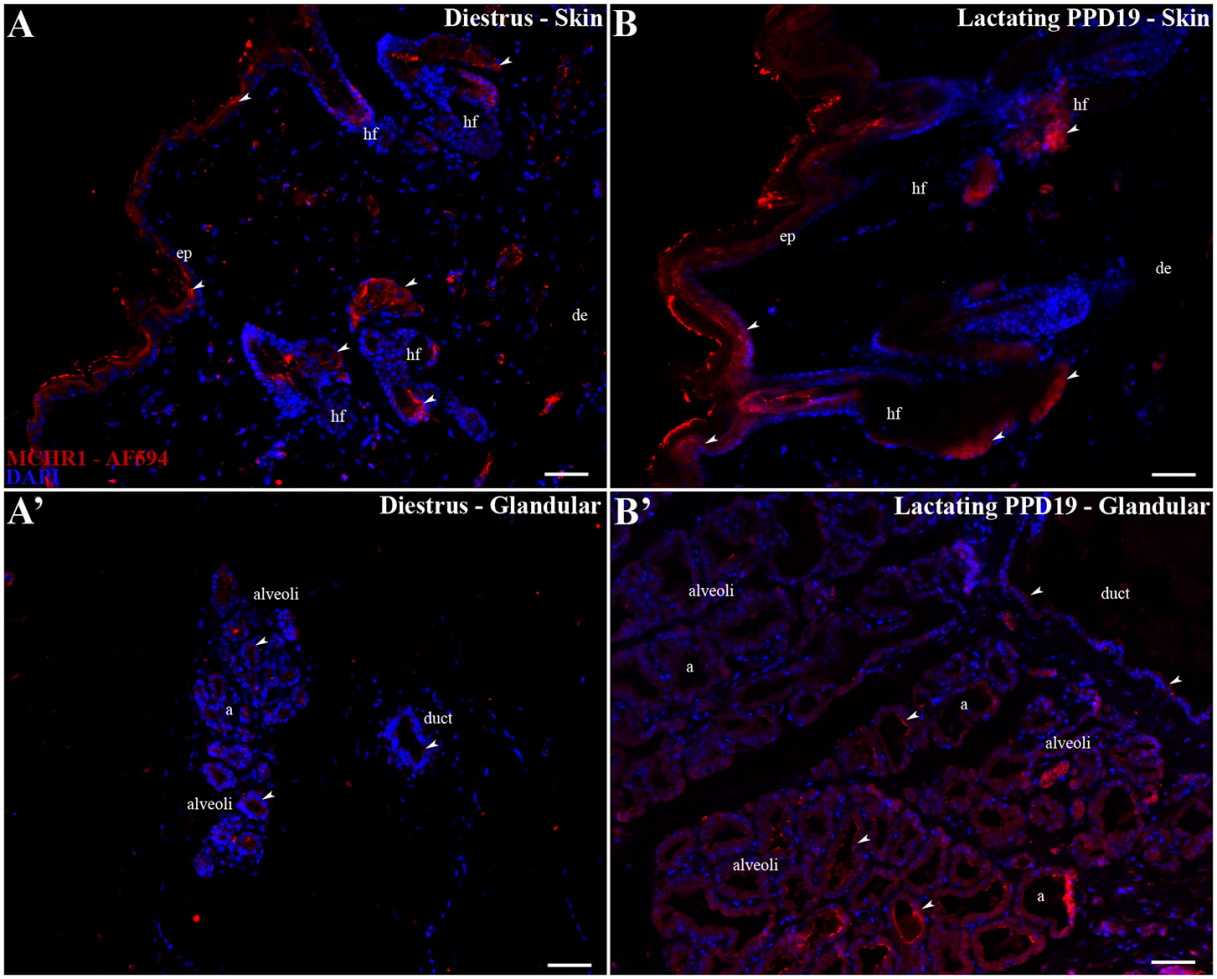
MCHR1 immunoreactivity in the skin and parenchyma of the rat mammary gland tissue. Wide field fluorescence photomicrographs of sagittal mammary gland slices from diestrus and PPD19 rats (*n* = 3/group) submitted to indirect immunofluorescence for MCHR1 (red) and counterstained with DAPI nuclear stain (blue). **(A,B)** In the skin of mammary gland the MCHR1-ir cells are found in the epidermis, distributed all over the basal, spinous, granular layers, and cornified layer. Some of the accessory epidermal structures of the skin were labeled, such as the fat cells of the sebaceous gland and cells of the hair follicle. **(A')** The undeveloped parenchyma on diestrus phase shows few or absence immunolabeling for MCHR1. **(B')** The parenchyma on PPD19 display MCHR-ir cells bordering and into the luminal part of the *acini* and ducts. The MCHR1-ir cells are indicated by white arrowheads. a, *acini*; ep, epidermis; de, dermis; hf, hair follicle; d, duct. Scale bar: 50 μm.

As first revealed by ISH, the skin overlying the mammary gland of diestrus and lactating rats is richly labeled for MCHR1, including cells located in the epidermis, distributed all over the basal, spinous, granular layers, and some MCHR1-ir labeled cells found very close to the cornified layer (Figures 3A,B). Some of the accessory epidermal structures of the skin were also labeled, such as the fat cells of the sebaceous gland and cells of the hair follicle (Figures 3A,B). Although the cells of *alveoli* and ducts located in the undeveloped parenchyma of the mammary gland of diestrus rats show few or absence immunostaining for MCHR1 (Figure 3A'), lactating dams display MCHR1-ir cells in the subjacent layers of developed parenchyma, such as muscle, adipose, and glandular tissues (Figure 3B').

**Figure 4:**
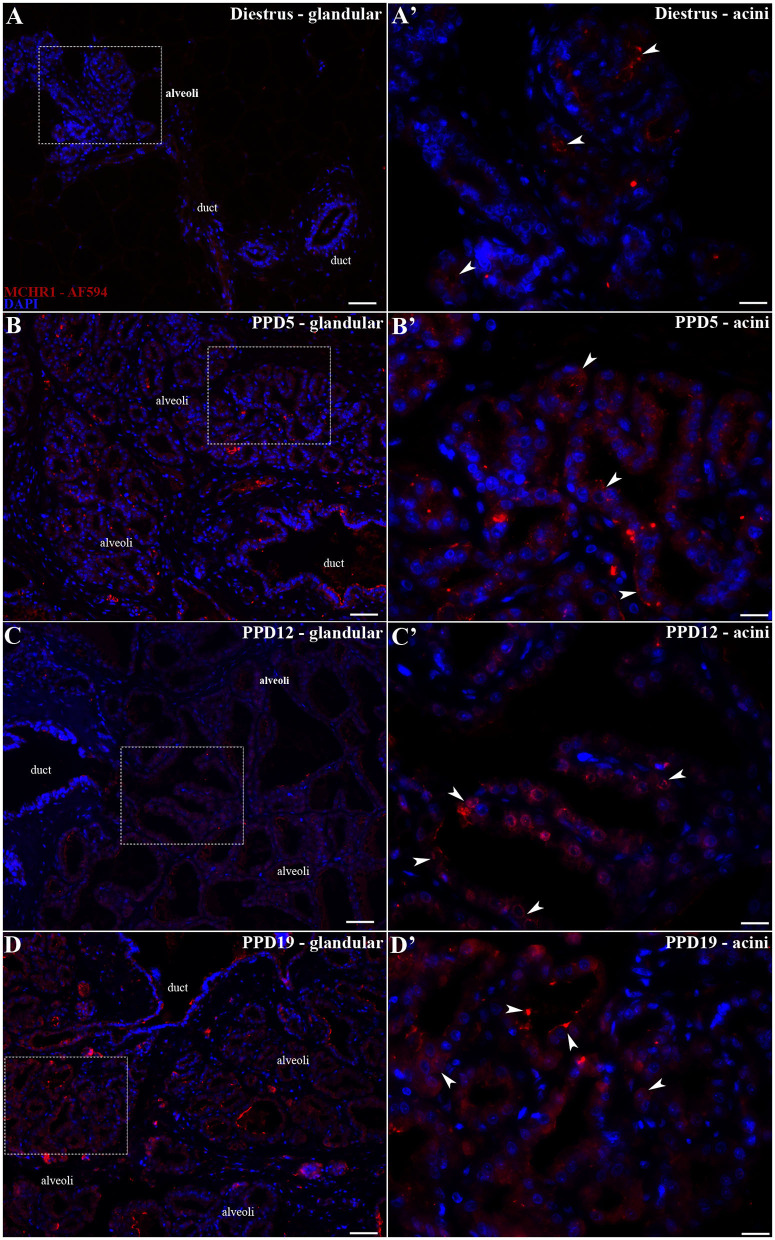
MCHR1 immunoreactivity in the rat mammary gland. Wide field fluorescence photomicrographs of sagittal mammary gland slices from diestrus and lactating rats (*n* = 3/group) submitted to indirect immunofluorescence for MCHR1 (red) and counterstained with DAPI nuclear stain (blue). **(A)** The mammary gland of diestrus, a tissue that is not totally differentiated as a lactating tissue, presents few MCHR1-ir cells. **(A')** Higher magnification of the square area in **(A)** showing few MCHR1-ir cells located mainly in islands of undifferentiated parenchyma of mammary gland tissue surrounded by adipose tissue. **(B–D)** Along the different time points of the lactation, there is an evident effect of lactation in the presence and location of MCHR1-ir cells in the rat mammary gland tissue. **(B'–D')** Higher magnification of the square areas in **(A–D)**. There is an increasing of MCHR-ir cells bordering and into the luminal part of the *acini* at PPD19 **(D')** when compared with the **(B')** PPD5 and **(C')** PPD12, which is similar with qPCR and Western blotting data. The MCHR1-ir cells are indicated by white arrowheads. Scale bars: 50 μm **(A–D)**, 20 μm **(A'–D')**.

The pattern of immunoreactivity was similar to that obtained through ISH: only sparse or absence immunolabeling was observed in the undeveloped parenchyma of diestrus rats (Figures 4A,A'). The labeled MCHR1-ir cells were found in the parenchyma located bordering the *acini* and in the ducts of the mammary *alveoli* along the lactation time points (Figures 4B–D). Moreover, we identified the presence of MCHR1-ir cells in the luminal part of the *acini* with an increasing pattern of distribution along and at the end of the lactation (Figures 4B'–D').

## Discussion

In this work, we report for the first time the presence of MCHR1 in the mammary gland of female rats and its correlation with the lactating status and postpartum period. Although a large body of information has been generated for MCHR1 within the central nervous system, little is known about this receptor action in the periphery despite the description of its mRNA in several tissues (11, 20, 23, 30). Although central MCH has been implicated in several functions [for review, see (38)], the actions of MCH in peripheral MCHR1 remain mostly unclear with the exception of a few works indicating a modulatory action of MCH over the immune system (49, 50).

Previous works have indicated a biphasic role for the MPOA synthesis of MCH in maternal behavior as MCH appears to be both necessary for the onset of maternal behavior and a deterrent to this behavior expression as lactation progresses (51–53). Our work indicates that MCH plays a similar role in milk secretion and mammary gland development in the postpartum period.

In this study, both *Mchr1* mRNA and MCHR1 immunoreactivity were found in the mammary glandular parenchyma. Within the parenchyma, MCHR1 was found codistributed with the *acini* (external layer), but it was most prevalent in the secretory cuboid cells lining the acinus lumen, mainly in lactating rats on PPD19. This double presence of MCHR1 in cells of the acinus could also represent a dual mechanism of MCH modulation of secretory and milk ejection function. Through myoepithelial cells, MCH can exert actions over milk ejection in synergistic way with OT since myoepithelial cells surrounding the mammary ducts contain OT receptors (54). At the same time, MCH may interact with the prolactin (PRL) system in the secretory cells given the presence of PRL receptors in this cell population. Prolactin binding to its receptors results in transcription of milk-specific proteins and inhibition of mammary involution (54–56). Given that PRL acts more as a survival factor than as a milk secretion–regulating factor (57), MCHR1 is located in a prime location to modulate both milk secretion and mammary gland maintenance.

A possible mechanism for peripheral MCHR1 activation is the release of MCH in the bloodstream, a phenomenon described by several authors in lactating and non-lactating contexts (11–16). The peak synthesis of MCH near the weaning period (6) and, consequently, the lactation end, and the anatomical basis for a mechanism of MCH release during this period advocate for a peripheral action of MCH over lactation cessation. Furthermore, the interaction between MCH and OT in the median eminence could be indicative of a role in milk synthesis/ejection modulation, which fits well with the thoroughly described action of MCH in orexigenic and energy expenditure processes (9, 35–37).

If this hypothesis is valid, it begs the question of what benefit would mammals have to have a secondary system acting with OT and PRL in the mammary acinus. When gene expression was quantified, we observed a biphasic pattern of *Mchr1* expression in lactating animals with PPD5 (early lactation) and PPD19 (late lactation) displaying the highest values. This mimics the biphasic role of MCH on maternal behavior, where a facilitating role on early lactation is superseded by a suppressive role toward PPD19. Transposing this to the mammary gland, it could indicate an increased *Mchr1* expression at the beginning of lactation to promote milk ejection, relaying visual, auditory, and olfactory stimuli perceived by the dam and integrated in the MPOA, possibly to modulate or anticipate the action of OT upon suckling stimulus. As the dam's energy reserves are depleted and the pups consume more milk, the homeostatic dampener role of MCH becomes the driving force, contributing to the cessation of lactation and involution of the mammary glands.

In unpublished data from our group, we verified the presence of MCH-ir cells in the same sites described here for the presence of MCHR1 in the mammary gland tissue of female rats. However, studies of molecular biology are needed to investigate local MCH synthesis in this tissue. Despite that, we cannot disregard the hypothesis that MCH is also synthesized locally, and it has autocrine and/or paracrine action on mammary gland tissue. Interestingly, there is evidence in the literature that support local production of MCH and its autocrine action as it was described for some other peripheral tissues, such as the endocrine pancreas, gastrointestinal tract, and testis (58–60), which supports the idea of the modulating role of MCH.

Our model fits well in the idea that pregnancy, lactation, and weaning are highly concerted central and peripheral actions. They are a physiological process that results in some of the most intense morphological and hormonal transformations an adult mammal can undergo in its lifetime. It is known that pregnant rats undergo central and peripheral alterations to cope with the nutritional demand of the pups (54, 61), guaranteeing its own homeostatic balance and adequate litter survival. Our data suggests that MCH is an important integrative player in this process, integrating external stimuli and the dam's own energy reserve to possibly modulate the major energetic sink during this period: milk production and ejection. However, more functional studies are necessary to completely clarify specific mechanisms underlying this process.

In summary, we have identified the rat skin and mammary gland parenchyma as new sites of *Mchr1* expression and MCHR1 synthesis. Although the presence of MCHR1 in the skin was independent of reproductive status, *Mchr1* expression in the mammary *acini* was temporally vinculated to the lactation stage. These results suggest that MCH released into the bloodstream can act to modulate physiological aspects of the mammary gland in addition to the canonical hormones OT and PRL, forming a class of unspecific modulators that act on the mammary gland to promote the coordination of milk ejection and mammary involution with the metabolic demands of the dam. Further studies are necessary to understand the role of the MCH/MCHR1 peptidergic system in the mammary gland to allow a better understanding about the mechanisms by which MCH acts in mammary tissue to modulate lactation-related events.

## Data Availability Statement

The raw data supporting the conclusions of this article will be made available by the corresponding author, without undue reservation, to any qualified researcher.

## Ethics Statement

The animal study was reviewed and approved by the Committee on Ethical Research on Animals of the Institute of Psychology (Protocol CEPA #003/2012) and by Ethics Committee on the Animal Use of the Institute of Biomedical Sciences (Protocol CEUA #035/2012).

## Author Contributions

DB: acquisition of the data, data analysis, interpretation, and final manuscript writing. AL, GD, LM-T, and MK: acquisition of the data, data analysis, and manuscript writing. JF: acquisition of the data. LS, JC-N, EB, PS, and JB: critical revision of the manuscript for important intellectual mentorship and content. CA, AA, JC-N, EB, PS, and JB: technical and material support. JB: study concepts and design, study supervision, and final manuscript writing. All authors had full access to the data, take responsibility for the data integrity, accuracy of the data analysis, final approval of the version to be published, and are responsible for the integrity of the work.

## Conflict of Interest

The authors declare that the research was conducted in the absence of any commercial or financial relationships that could be construed as a potential conflict of interest.

## Abbreviations

GPCRs: G-protein couple receptors
*Mchr1*: receptor 1 MCH, gene
MCHR1: receptor 1 MCH, protein
MCHR2: receptor 2 MCH, protein
MPOA: medial preoptic area
OT: oxytocin
*Pmch*: melanin-concentrating hormone, gene
PRL: prolactin.
