# Supplementary figures and images for: The Rat Mammary Gland as a Novel Site of Expression of Melanin-Concentrating Hormone Receptor 1 mRNA and Its Protein Immunoreactivity
Source: Front Endocrinol (Lausanne). 2020 Jul 31;11:463. doi: 10.3389/fendo.2020.00463 (PMC7411258; doi:10.3389/fendo.2020.00463)

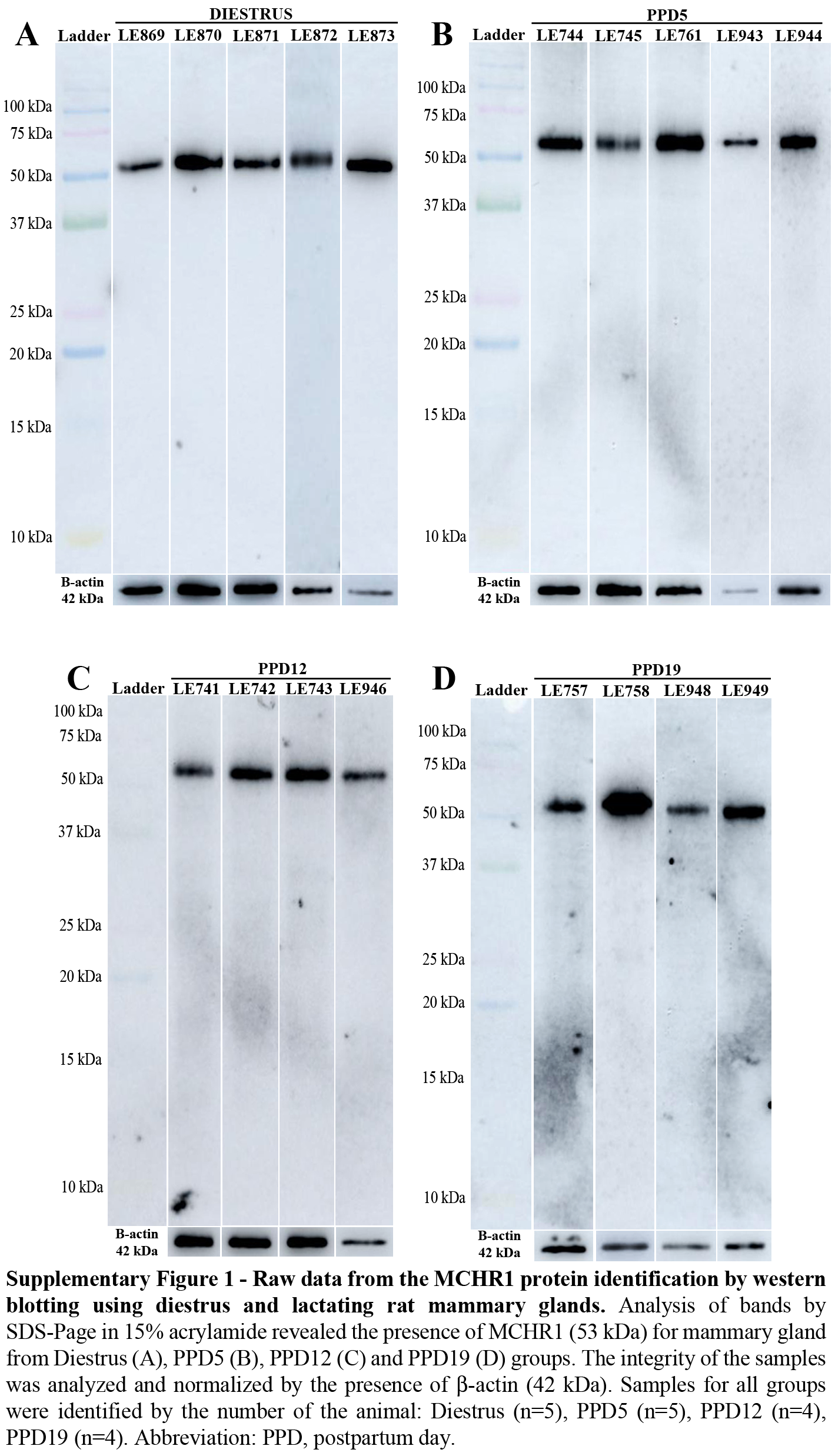

Supplement: Supplementary file 1 [file Image_1.TIF]

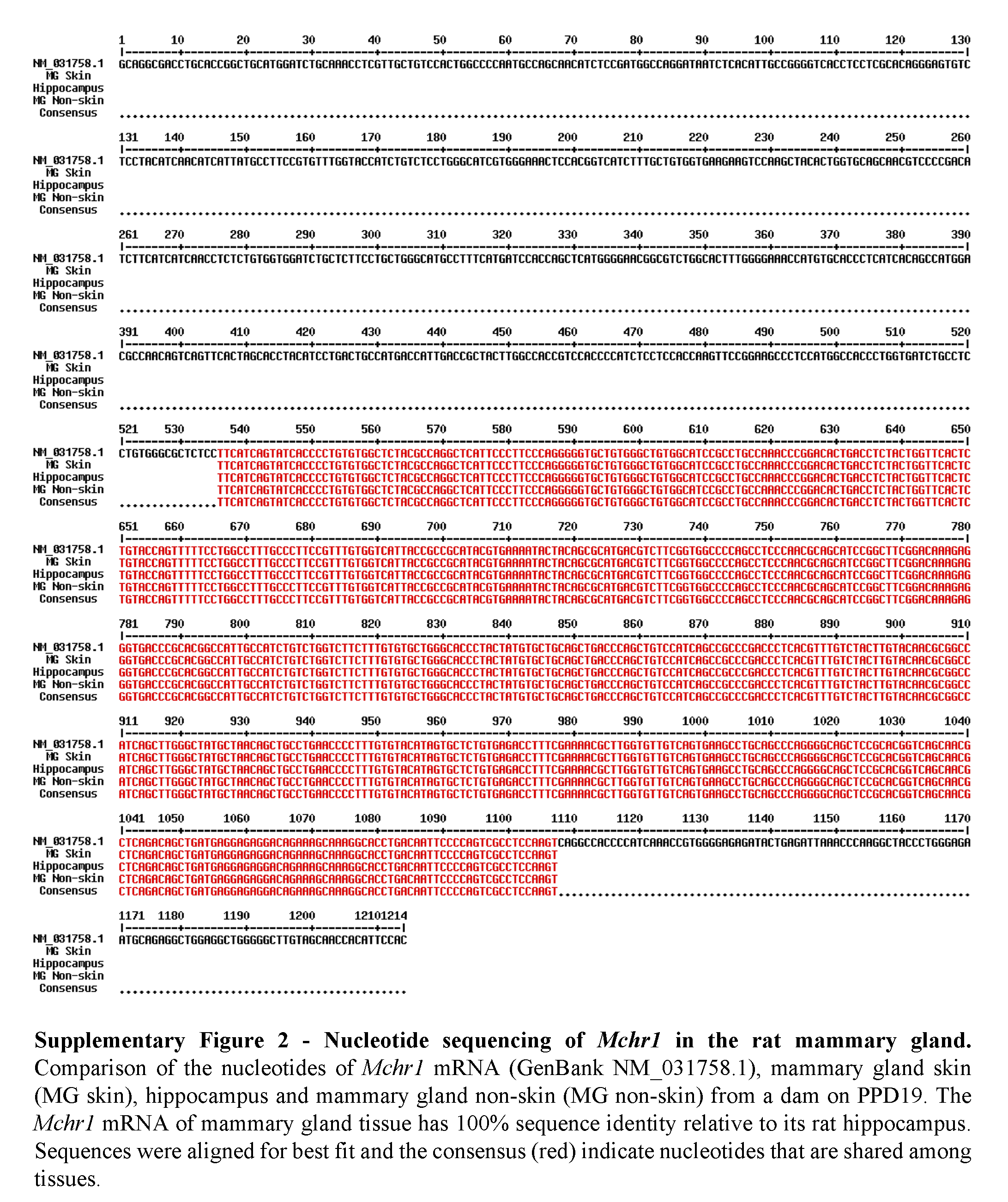

Supplement: Supplementary file 2 [file Image_2.TIF]

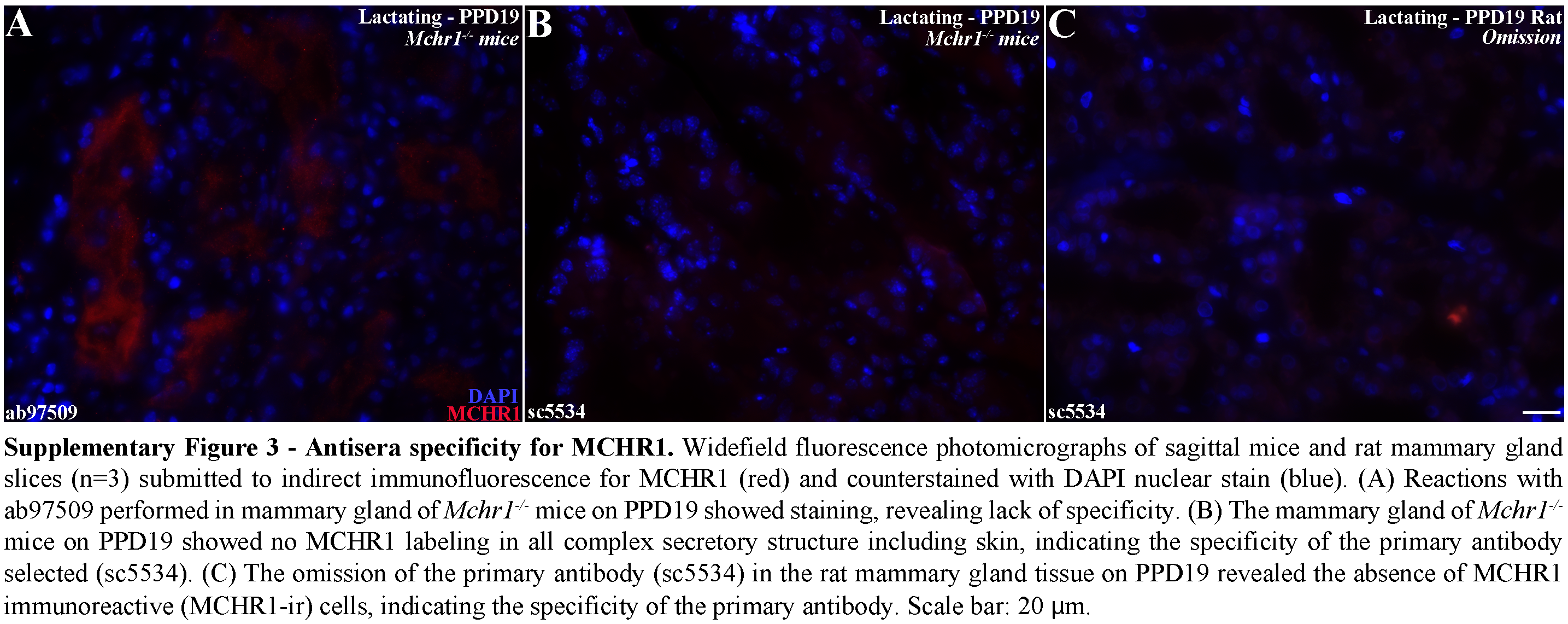

Supplement: Supplementary file 3 [file Image_3.TIF]
